# In Vivo Bactericidal Efficacy of GWH1 Antimicrobial Peptide Displayed on Protein Nanoparticles, a Potential Alternative to Antibiotics

**DOI:** 10.3390/pharmaceutics12121217

**Published:** 2020-12-17

**Authors:** Jose V. Carratalá, Eric Brouillette, Naroa Serna, Alejandro Sánchez-Chardi, Julieta M. Sánchez, Antonio Villaverde, Anna Arís, Elena Garcia-Fruitós, Neus Ferrer-Miralles, François Malouin

**Affiliations:** 1 Institute for Biotechnology and Biomedicine, Autonomous University of Barcelona, Bellaterra, 08193 Barcelona, Spain; JoseVicente.Carratala@uab.cat (J.V.C.); naroa.serna@uab.cat (N.S.); jsanchezqa@gmail.com (J.M.S.); antonio.villaverde@uab.cat (A.V.); 2 Department of Genetics and Microbiology, Autonomous University of Barcelona, Bellaterra, 08193 Barcelona, Spain; 3 Bioengineering, Biomaterials and Nanomedicine Networking Biomedical Research Centre (CIBER-BBN), C/Monforte de Lemos 3-5, 28029 Madrid, Spain; 4 Centre d’Étude et de Valorisation de la Diversité Microbienne (CEVDM), Département de Biologie, Université de Sherbrooke, 2500 Boul. Université, Sherbrooke, QC J1K 2R1, Canada; eric.brouillette@usherbrooke.ca; 5 Mastitis Network and Regroupement de Recherche Pour un Lait de Qualité Optimale (Op+Lait), Université de Montréal, 2900 Edouard Montpetit Blvd, Montréal, QC H3T 1J4, Canada; 6 Microscopy Service, Autonomous University of Barcelona, Bellaterra, 08193 Barcelona, Spain; Alejandro.Sanchez.Chardi@uab.cat; 7 Departament of Evolutionary Biology, Ecology and Environmental Sciences, University of Barcelona, Avda Diagonal 643, 08028 Barcelona, Spain; 8 Department of Ruminant Production, Institute of Agriculture and Agrifood Research and Technology (IRTA), Caldes de Montbui, 08140 Barcelona, Spain; anna.aris@irta.cat (A.A.); elena.garcia@irta.cat (E.G.-F.)

**Keywords:** mouse mastitis model, antimicrobial peptide, protein nanoparticle, inclusion body, recombinant protein, *Escherichia coli*, *Staphylococcus aureus*, therapeutic protein

## Abstract

Oligomerization of antimicrobial peptides into nanosized supramolecular complexes produced in biological systems (inclusion bodies and self-assembling nanoparticles) seems an appealing alternative to conventional antibiotics. In this work, the antimicrobial peptide, GWH1, was N-terminally fused to two different scaffold proteins, namely, GFP and IFN-γ for its bacterial production in the form of such recombinant protein complexes. Protein self-assembling as regular soluble protein nanoparticles was achieved in the case of GWH1-GFP, while oligomerization into bacterial inclusion bodies was reached in both constructions. Among all these types of therapeutic proteins, protein nanoparticles of GWH1-GFP showed the highest bactericidal effect in an in vitro assay against *Escherichia coli*, whereas non-oligomerized GWH1-GFP and GWH1-IFN-γ only displayed a moderate bactericidal activity. These results indicate that the biological activity of GWH1 is specifically enhanced in the form of regular multi-display configurations. Those in vitro observations were fully validated against a bacterial infection using a mouse mastitis model, in which the GWH1-GFP soluble nanoparticles were able to effectively reduce bacterial loads.

## 1. Introduction

Novel antimicrobial and engineering approaches are urgently needed to cope with antibiotic resistance. Antimicrobial peptides (AMPs), a class of naturally occurring small (generally less than 50 amino acids) and positively charged peptides [1], have attracted attention in clinical research because of their broad-spectrum activity against a diverse group of microorganisms, including antibiotic-resistant pathogens [2]. On the other hand, cytokines are a group of small immunomodulatory proteins that play a central role in host defense by orchestrating the antimicrobial functions and conferring greater protection against different infectious agents [3]. One of the most studied cytokines is interferon-gamma (IFN-γ), which has proven to be a potent immunoprophylactic agent [4,5].

However, under physiological conditions, AMPs are subjected to proteolytic degradation and peptide inactivation by nonspecific interactions with anionic substances, which result in the low bioavailability and poor in vivo stability of these small molecules [6]. Furthermore, most cytokines, including IFN-γ, have very short half-lives, so their immunological function is limited [3]. Therefore, the accumulation of these proteins in naturally occurring bacterial inclusion bodies (IBs) and soluble self-assembling protein-only nanoparticles (PNPs) seems an appealing alternative to overcome these limitations. In 2017, Serna et al. described for the first time the use of PNPs as antibacterial agents [7]. These soluble PNPs were obtained following a modular protein design based on the fusion of a cationic peptide to a C-terminal his-tagged scaffold protein [8]. This modular configuration was presented as a transversal platform which has been replicated with several scaffold proteins [9,10]. The cationic α-helical GWH1 antimicrobial peptide [11], once fused to the amino terminus of green fluorescent protein (GFP), promoted the oligomerization in PNPs of around 50 nm size which showed a wide bactericidal effect against different pathogenic bacteria in cell cultures [7]. Due to the functional and structural versatility of this system, we wondered whether the fusion design between GWH1 and IFN-γ could also lead to the formation of these highly stable nanosized oligomers (PNPs). Besides, bacterial IBs, once considered as waste by-products derived from recombinant protein production, now provide a useful source of ready-to-use active protein. Inside these structures, therapeutic proteins are stored in native and native-like conformations and are released under physiological conditions [12,13,14]. The benefits of this system lie in the protective effect against degradation and the sustained release of the protein, which in both cases can significantly increase their half-lives.

The aim of the present study is to characterize and to evaluate, in an in vivo mastitis mice model, the direct and non-direct antibacterial effects of two different protein designs (GWH1-GFP and GWH1-IFN-γ) assembled in two protein formats, namely, PNPs and IBs. Furthermore, we wanted to test the synergy of both activities, immunomodulation and bactericidal effect, in a single polypeptide with the potential to form PNPs. The results obtained here could throw some light on the development of new protein-based mastitis therapies but with transversal applicability in any clinical problem needing unconventional antimicrobial therapies.

## 2. Materials and Methods

### 2.1. Protein Design and Production

All DNA sequences (GWH1-GFP, GWH1-IFN-γ, IFN-γ and GFP) were codon optimized (*Escherichia coli*), synthetized and cloned in pET22b plasmids by GeneArt (Waltham, MA, USA). IFN-γ from mouse origin was selected in sequence designs due to the species-specific nature of this protein. *E. coli* BL21 (DE3) (Novagen, Madison, WI, USA) was chosen as expression host. Protein production was performed in 2 L volume flasks containing 500 mL of LB medium with 100 µg/mL ampicillin (plasmid resistance). Cells were incubated at 37 °C until the culture reached an optical density of around 0.5, at this point 0.1 mmol/L of isopropyl β-D-1-thiogalactopyranoside (IPTG) was added to each culture and expression temperature was set at 20 °C for 5 h. For IBs, production times were established for 3 h at 37 °C. Cells were harvested by centrifugation (20 min, 5000 rpm) and stored at −80 °C.

### 2.2. Protein Purification

**Scheme 1:**
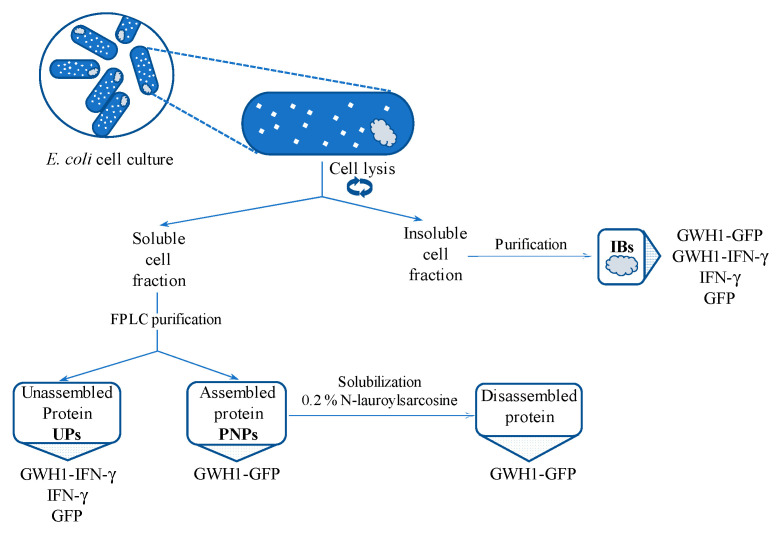
Schematic representation of the protein formats used through the study. During protein production, recombinant protein was accumulated inside *E. coli* cells as insoluble aggregates (inclusion bodies—IBs) or in a soluble state. After cell lysis and centrifugation, IBs were recovered from the cell debris. Supernatant was used for soluble protein purification purposes. Positive protein peaks were analyzed by dynamic light scattering (DLS) and protein samples were categorized as unassembled protein versions or assembled protein versions. Assembled protein GWH1-GFP was transformed into disassembled protein by incubation with mild detergent. The names of the protein samples used in the mastitis mice model are labeled beside or below the protein versions enclosed in a light blue box.

For soluble protein, purification procedures were performed as described before [9], with some modifications. The cell pellet was resuspended in wash buffer (Tris 20 mmol/L, pH 8.0, NaCl 500 mmol/L, imidazole 10 mmol/L) with DNase (1 mg/mL) (Thermo-Scientific, Waltham, MA, USA) and ethylenediamine tetra-acetic acid-free protease-inhibitor (complete EDTA-Free, F. Hoffmann-La Roche AG, Basel, Switzerland). French press was selected as disruption method for cell lysis (3 rounds at 1200 psi). The protein amount was quantified by Bradford, aliquoted and stored at −80 °C for further experiments. A schematic representation of the different types of bioactive materials obtained after protein purification is outlined in Scheme 1. Purified recombinant protein from the soluble cell fraction by affinity chromatography generated two types of protein samples depending on macromolecular organization (unassembled and assembled proteins (PNPs)). Purified proteins were stored in sodium bicarbonate buffer containing 166 mmol/L NaHCO_3_ and 333 mmol/L NaCl. Bacterial IBs were purified and quantified as described earlier [15]. An additional step of French Press disruption (3 rounds at 1200 psi) was included at the beginning of the process. Sterile conditions were maintained throughout the process. Pelleted IBs were resuspended in sodium bicarbonate buffer prior to its use. Purification productivity of analyzed recombinant proteins as well as protein solubility is detailed in Supplementary Table S1.

### 2.3. Nanoparticle Size Characterization

Size distribution of protein samples was determined by dynamic light scattering (DLS). Average values were obtained after the independent measurement of protein samples in triplicates at 633 nm in a Zetasizer Nano ZS (Malvern Instruments Ltd., Malvern, UK). Nanoscale morphological characterization of isolated IBs was performed with two high resolution electron microscopy techniques adapted to this type of biomaterial [16,17,18,19,20,21,22,23]. Sample preparation for the field emission scanning electron microscope (FESEM) observation was described previously [12]. Pellets of the same four isolated IBs were processed following conventional transmission electron microscopy (TEM) procedures as previously described [16,17].

### 2.4. Bactericidal Activity Assay

The effect of the different antimicrobial candidates was evaluated against *E. coli* ATCC 25922 and *Staphylococcus aureus* ATCC 29737. The assay was performed using a broth micro-dilution method. In 96-well plates, after a two-fold dilution process in 50 µL, each well contained a specific amount of the corresponding protein, ranging from 6 to 0.4 µmol/L. After protein distribution, 50 µL of Mueller Hinton Broth Cation-adjusted medium (MHB-II, Sigma-Aldrich, Saint Louis, MO, USA) containing 10^6^ CFU/mL (colony forming units per mL) of *E. coli* or *S. aureus* was inoculated in each well. Maximal growth was achieved in control wells with no protein. Each concentration was evaluated in duplicates. After microorganism inoculation, the 96-well plates were gently agitated and then incubated without agitation at 37 °C for 18 h. Bacterial viability was evaluated using the commercially available BacTiter-Glo™ Microbial Cell Viability Assay (Promega, Madison, WI, USA) following the manufacturer’s instructions. Luminescence was measured using the Multilabel Plater Reader VICTOR3 (PerkinElmer, Waltham, MA, USA).

### 2.5. Disassembling of Soluble Nanoparticles

GWH1-GFP PNPs were disassembled after the addition of the mild-solubilization agent N-lauroylsarcosine 0.2% (Sigma-Aldrich, Saint Louis, MO, USA) (see Scheme 1). The disassembling process was evaluated by DLS at 633 nm in a Zetasizer Nano ZS (Malvern Instruments Ltd., Malvern, UK). Bactericidal activity of assembled and disassembled GWH1-GFP proteins was evaluated as described in Section 2.4, apart from the protein concentration range, that in this case ranged from 0.5 to 8 µmol/L.

### 2.6. Protein Solubilization from IBs

The different protein IBs (GWH1-GFP, GWH1-IFN-γ, IFN-γ and GFP) were resuspended in 250 µL of sodium bicarbonate buffer. The conditions for solubilization were established at 37 °C for 18 h with agitation. After the incubation, the solubilized protein in the supernatant was recovered by centrifugation (15 min, 15,000× *g*). Size distribution of the solubilized protein was evaluated by DLS and the amount of protein released from the IBs was determined by Western blot, as detailed [12].

### 2.7. Determination of Murine IFN-γ Biological Activity

The procedure for the activity evaluation of IFN-γ was adapted from a nitric oxide (NO)-based bioassay [24]. Detailed modifications are described as follows. Serial dilutions of the different IFN-γ constructs (commercial *E. coli*-derived murine IFN-γ (R&D systems, Minneapolis, MN, USA), recombinant in-house murine IFN-γ and GWH1-IFN-γ) at quantities ranging from 6 to 120 nmol/L were incubated with murine microglial cells (BV-2) for 48 h at 37 °C. No protein was added in control wells. To study the specificity of the NO response, a specific concentration (120 nmol/L) of a commercial recombinant mouse IFN-γ was evaluated in the absence and presence of 500 µmol/L L-NMA (Sigma-Aldrich, Sant Louis, MO, USA), an inhibitor of nitric oxide synthase (i-NOS).

### 2.8. Mouse Model of Infectious Mastitis

The mouse mastitis model was previously described [25,26,27]. CD-1 lactating micewere used. Experiment specifications are briefly reported here. A total of 100 µL of bacteria (100 CFUs of either *E. coli* ATCC 25922 or *S. aureus* ATCC 29737) and 100 µL of the corresponding protein compound (assembled PNPs, unassembled soluble protein or IBs) were administered in each mammary gland pair. A minimum of three mice (6 mammary glands) were employed to test each protein compound. Protein concentrations in all cases were adjusted to 60 µmol/L after dilution (unassembled versions and PNPs) or resuspension (IBs) with sodium bicarbonate buffer with salt. In experimental controls, 100 µL of sodium bicarbonate buffer with salt was administered instead of the protein-containing solutions. At relevant time points post-inoculation (6, 12 and 24 h for kinetics growth studies and 8 h for compound efficacy studies), mice were sedated and euthanized. Bacterial CFU contained in samples were evaluated by plating serial dilutions of gland homogenates on tryptic soy agar (TSA) plates and raw CFU counts converted into base 10 logarithm values. The animal experiments were performed in accordance with the guidelines of the Canadian Council on Animal Care and approved by the ethics committee on animal experimentation of the Faculté des Sciences of Université de Sherbrooke (protocol 2017–1966 FM2017-01B).

### 2.9. Statistical Analysis

All statistical analyses were conducted in GraphPad Prism version 8.0.0 for Windows (GraphPad Software, San Diego, CA, USA) with at least three independent replicates unless otherwise indicated. Bars in figures are expressed as mean ± standard deviation. Quantitative data were tested for normality and equality of variances with Kolmogorov–Smirnov and Levene tests, respectively, prior to analyses. Depending on the experiment, statistical divergences were evaluated using one-way ANOVA with Tukey’s multiple comparisons test or two-tailed unpaired Student’s *t* test. Statistical significance in relevant results is indicated in figures as * *p* < 0.05, ** *p* < 0.01, and *** *p* < 0.001.

## 3. Results

### 3.1. Characterization of Self-Assembling Nanoparticles

**Figure 1:**
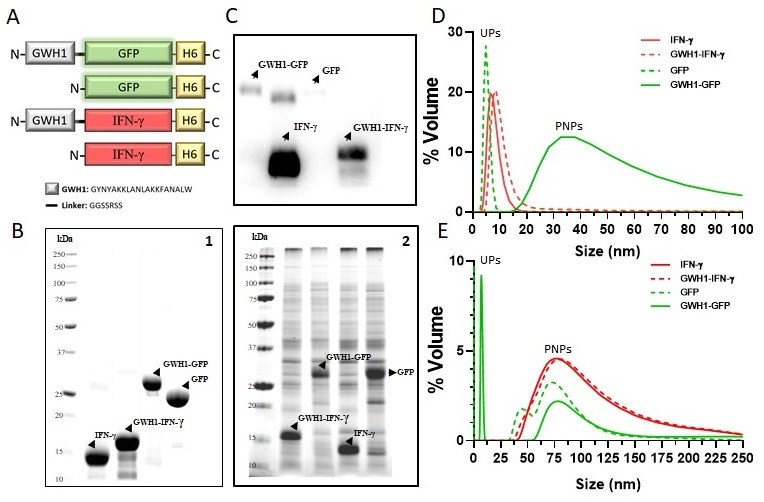
Characterization of the different protein formats employed in this study. (**A**) Schematic representation of multimeric fusion proteins. The presence of a cationic peptide (GWH1) in the N-terminus, plus the His6 tag in C-terminus, may induce the formation of protein-only nanoparticles (PNPs). A linker between GWH1 and the scaffold protein was added in order to increase flexibility and facilitate self-assembling process. (**B**) TGX Stain-free^TM^ analysis showing the purity of the different protein designs in both, soluble proteins (**B1**) and IBs (**B2**). (**C**) Western blot showing the qualitative amount of protein released from the different IBs. (**D**) Evaluation of self-assembling capabilities of purified soluble proteins by size determination using DLS. PNPs: protein nanoparticles; UPs: unassembled proteins. (**E**) Size distribution of the protein samples obtained after solubilization from different IBs analyzed by DLS.

In the modular protein designs, GWH1 was N-terminally fused to two different his-tagged protein scaffolds (Figure 1A), namely, the previously reported GWH1-GFP [7] and the new design, GWH1-IFN-γ. After purification of recombinant proteins accumulated in the soluble cell fraction, high purity was reached with all protein designs (Figure 1(B1)). Self-assembling of monomers into soluble protein nanoparticles (PNPs) is driven by the fusion of a cationic peptide (GWH1) with His6-tagged scaffolds [10]. However, under the tested experimental conditions, protein oligomerization in soluble PNPs was only achieved with GWH1-GFP (Figure 1D) as previously described [7]. In DLS analysis, the peak size for this oligomer was around 35 nm. The high polydispersity showed by the sample could indicate the presence of a diverse mixture of PNPs with different oligomeric states (Figure 1D). Modular protein GWH1-IFN-γ showed an average size of around 10 nm (Figure 1D). When compared to IFN-γ, only a slight difference in size was observed, suggesting that protein oligomerization was not achieved with this fusion protein design. As expected, GFP and IFN-γ did not form PNPs (Figure 1D).

### 3.2. In Vitro Evaluation of Antibacterial Activity

**Figure 2:**
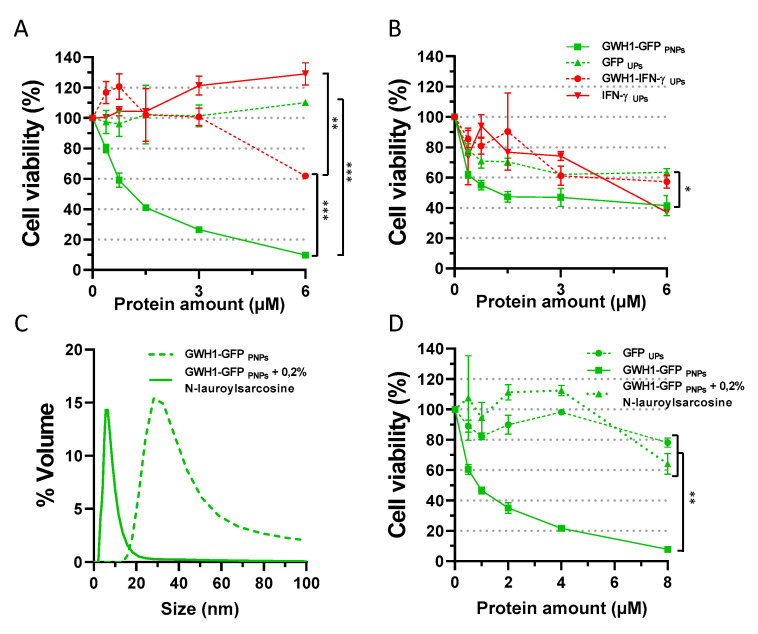
Antimicrobial activity of assembled (PNPs) and unassembled soluble proteins (UPs) in vitro. (**A**) Effect on cell viability of increasing amounts of GWH1-GFP and GWH1-IFN-γ against *E. coli* and (**B**) *S. aureus* (**C**). Dynamic light scattering (DLS) analysis showing the nanoparticle disassembling of GWH1-GFP in presence of 0.2% N-lauroylsarcosine. (**D**) Comparative effect on cell viability of disassembled (promoted in presence of 0.2% N-lauroylsarcosine) and assembled GWH1-GFP against *E. coli* in vitro. IFN-γ and GFP were used as controls. Significant results are shown as * *p* ≤ 0.05, ** *p* ≤ 0.01 and *** *p* ≤ 0.001, multiple *t* test. All experiments were performed in duplicates. Bars represent the the mean ± standard deviation.

The antimicrobial potential of soluble GWH1-GFP (PNPs) and soluble GWH1-IFN-γ (unassembled protein (UP) forms) was evaluated in an in vitro assay against two of the most common pathogens involved in mastitis, *S. aureus* and *E. coli*. GFP and IFN-γ were used as controls for their antimicrobial peptide carrier counterparts. GWH1-GFP showed a dose-dependent antibacterial activity against *E. coli* reducing cell viability by up to 90% (Figure 2A). On the other hand, GWH1-IFN-γ UPs did not follow the same pattern, only showing antibacterial activity at the highest concentration, reducing bacterial viability by 40%. As expected, GFP and IFN-γ had no significant effect on cell viability (Figure 2A). On *S. aureus*, a dose-dependent pattern was observed again with GWH1-GFP PNPs (Figure 2B). However, its effect on cell viability was not as pronounced as that observed with *E. coli*. GWH1-IFN-γ UPs had a similar effect, reducing cell viability by 40% at the highest concentration. Control proteins, GFP and IFN-γ, showed an unspecific effect on cell viability, reducing bacterial loads to the same extent as that observed for the GWH1 carrier versions (Figure 2B).

### 3.3. Importance of Nanoparticle Format on Antibacterial Activity

Given that the nanoparticulated format seems to properly display the antimicrobial activity, the PNPs formed by GWH1-GFP were incubated with a mild solubilization agent (N-lauroylsarcosine) in order to disassemble the oligomeric format while preserving the native-like protein structure [28]. The presence of monomers of 5 nm size after achieving the final concentration of 0.2% N-lauroylsarcosine was observed by DLS (Figure 2C). In *E. coli*, the monomeric version of GWH1-GFP lacked the dose-dependent antimicrobial behavior showed by the PNP version, only acting at the highest concentration by reducing cell viability by 40%, a low antimicrobial effect compared with its fully active version, which reduced viability by more than 90% (Figure 2D).

### 3.4. Bioactivity Evaluation of IFN-γ-Based Proteins

**Figure 3:**
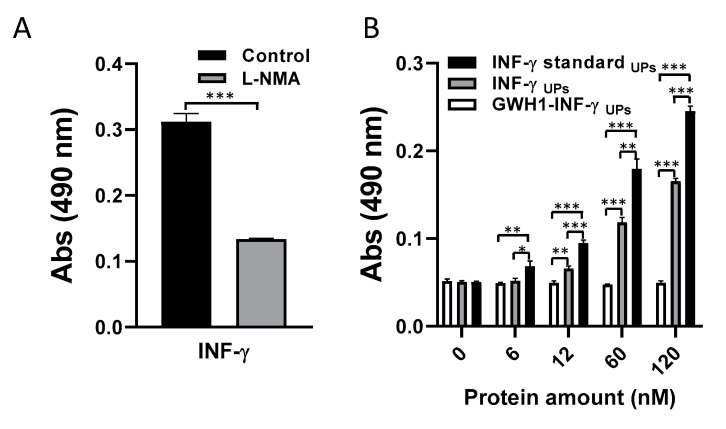
Nitric oxide (NO)-based bioassay for mouse IFN-γ activity evaluation in the murine microglial cell line BV-2. (**A**) Inhibitory effect of L-NMA (NG-monomethyl-L-arginine) on nitric oxide production by BV-2 in presence of a commercial recombinant mouse IFN-γ (120 nmol/L). Significant results are shown as *** *p* ≤ 0.001, unpaired Student’s *t* test. (**B**) Nitric oxide production (measured by its absorbance at 490 nm) for increasing amounts of the different IFN-γ-based constructs using, as standard, a commercial recombinant mouse IFN-γ. Significant results are shown as * *p* ≤ 0.05, ** *p* ≤ 0.01 and *** *p* ≤ 0.001, multiple *t* test. All experiments were performed in triplicates and data are represented as mean ± standard deviation.

The measurement of nitric oxide (NO) produced by the murine microglial cell line BV-2, in the presence of IFN-γ, provides a simple method for detection of bioactive mouse IFN-γ. The use of a selective inhibitor of iNOS, NG-monomethyl-L-arginine (L-NMA) allows one to evaluate the specificity of the response. An inhibition in the NO production by BV-2 cells was observed when L-NMA was added in the presence of commercial mouse IFN-γ (Figure 3A). The activity of the two mouse IFN-γ designs (IFN-γ and GWH1-IFN-γ UPs) was compared to a standard calibrated mouse IFN-γ. BV-2 cells produced nitrite in a dose-dependent manner in response to IFN-γ at protein concentrations between 6 and 120 nmol/L. The biological activity of the standard mouse IFN-γ was significantly higher than that of the recombinant IFN-γ produced in this study. Surprisingly, GWH1-IFN-γ UPs did not show any response in that range of concentrations, while recombinant in-house INF-γ was effective from 12 nmol/L (Figure 3B).

### 3.5. Growth Kinetics of E. coli in the Mastitis Mice Model

**Figure 4:**
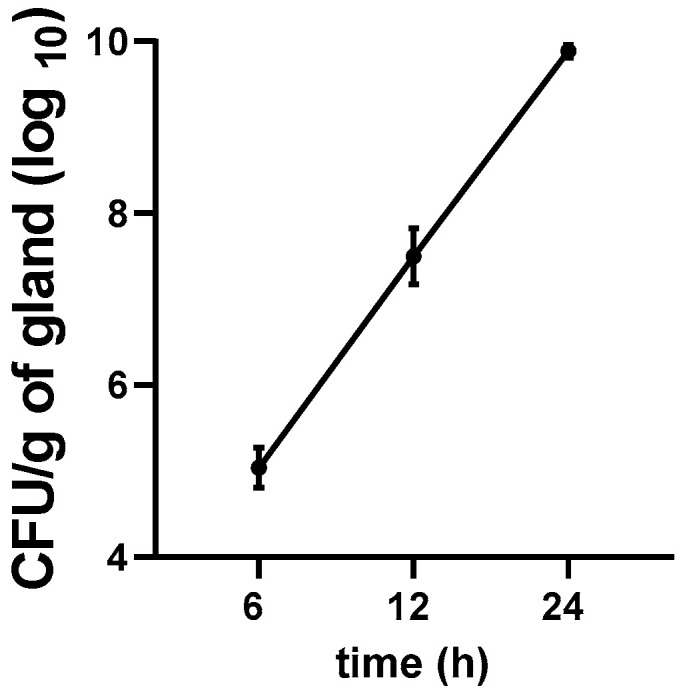
Growth kinetics of *E. coli* in the mouse mammary gland at 6, 12 and 24 h after the administration of 100 CFU through the teat channel. The bacterial loads for each time point were evaluated in 8 individual mammary glands (4 mice).Bars represent the mean ± standard deviation.

The intramammary infection (IMI) was monitored in a time course experiment (Figure 4). The medians of bacterial loads were 1.2 × 10^5^ CFU, 3.8 × 10^7^ CFU and 7.8 × 10^9^ CFU per g of gland after 6, 12 and 24 h, respectively. Results demonstrated a steady growth and since higher bacterial loads affected the general health of the animals, an infection time of 8 h was selected as the experimental condition for therapeutic efficacy studies.

### 3.6. Characterization of IBs

**Figure 5:**
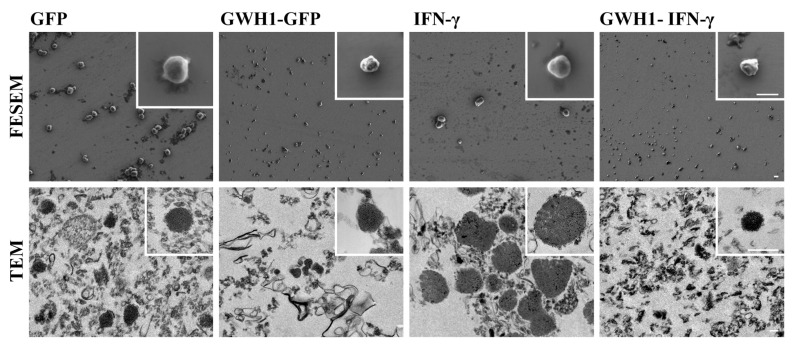
Ultrastructural characterization and analysis by electronic microscopy of purified inclusion bodies. Representative images of general fields and nanoparticle detail of entire IBs of GFP, GWH1-GFP, IFN-γ and GWH1-IFN-γ at two different magnifications, 11,000×/150,000× (FESEM) and 3000×/10,000× (TEM). Magnifications are equivalent in each micrograph to allow comparative visualization. The scale bars represent 500 nm.

Recombinant protein production in bacteria induces the formation of discrete protein clusters usually located at the cellular poles. These supramolecular complexes, known as IBs, contain functional recombinant protein. Released recombinant proteins tend to co-purify with other cellular proteins using IBs purification methods. In any case, in this study, a representative amount of the produced recombinant protein was observed in all cases (Figure 1(B2)). IFN-γ IBs showed the best releasing efficiency, being the amount of protein observed qualitatively different from the other candidates (Figure 1C). On the other hand, just a faint band was observed for GFP-based proteins, indicating a poor releasing process (Figure 1C). The size distribution of the solubilized protein was also evaluated (Figure 1E). Although soluble aggregates were mainly released from all protein designs, only GWH1-GFP included a monomeric distribution, which indicated the presence of two populations of different sizes. The ultrastructural characteristics of the IBs were analyzed by electronic microscopy (EM) (Figure 5). The results showed the presence of round-shaped IBs with a similar electrodensity in all samples but with marked differences between GWH1-based IBs and the GWH1-free (IFN-γ and GFP) IBs (Figure 5). Interestingly, in *E. coli*, IFN-γ formed a high amount of IBs with similar size and shape than GFP IBs. Conversely, GWH1-GFP and GWH1-IFN-γ IBs showed a shape–size distribution completely different from those observed for the well-formed GFP IBs. In addition, a high variability in size can be observed between these samples, suggesting the presence of not completely formed IBs (Figure 5).

### 3.7. Therapeutic Effect of PNPs and IBs in the Mouse Mastitis Model

**Figure 6:**
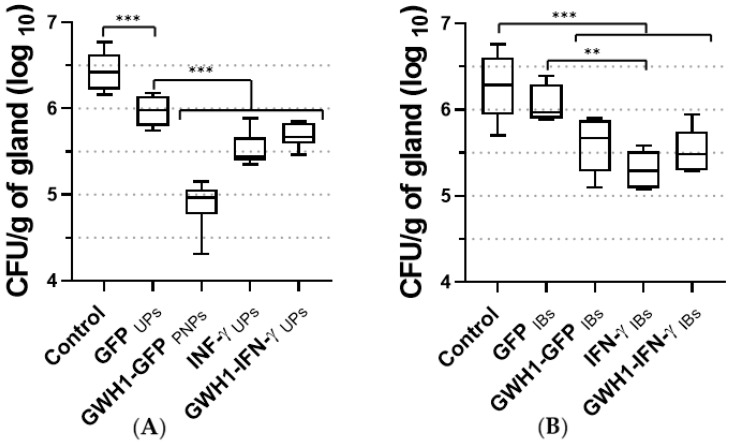
Bacterial loads (*E. coli* CFU/g of gland log10) measured after the administration of therapeutic and non-therapeutic protein formats into infected mammary glands. (**A**) Effect of unassembled protein forms (UPs) and assembled protein-only nanoparticles (PNPs) 8 h after the challenge with 100 CFU of *E. coli*. (**B**) Effect of the inclusion body (IB) format 8 h after the challenge with 100 CFU of *E. coli*. In all cases, 60 µM of the different protein formats were administered just after the infection with *E. coli*. Sodium bicarbonate buffer (166 mmol/L NaHCO_3_, 333 mmol/L NaCl) and GFP were used as controls. The middle bars indicate median values for each group of glands (at least 6 glands) whereas the boxes specify quartiles Q1-Q3. Experiments were performed with at least three independent replicates. Significant results are shown as ** *p* ≤ 0.01 and *** *p* ≤ 0.001, one-way analysis of variance (ANOVA) followed by Tukey’s multiple comparisons test.

A single dose of 60 µmol/L of each different protein design, in both soluble (UPs or PNPs) and insoluble (IBs) formats, was administered just after the intramammary inoculation of *E. coli*. The soluble PNPs of GWH1-GFP and the soluble UP versions of IFN-γ and GWH1-IFN-γ provoked a significant decrease in bacterial loads in comparison to both the control (sodium bicarbonate buffer) or GFP treatments (Figure 6A). The presence of GFP had, however, some negative effect on *E. coli* growth (Figure 6A). IFN-γ and GWH1-IFN-γ UPs caused a decrease in bacterial loads by 2.8 and 2 times greater than that caused by GFP, respectively. Nevertheless, the strongest bactericidal effect was obtained by the nanoparticle-forming protein GWH1-GFP, which decreased the bacterial loads by 35 times in comparison to the control treatment and by 11.5 times compared with the GWH1-free GFP. On the other hand, the same protein designs produced as IBs showed a different outcome (Figure 6B). The GFP IBs had a similar effect on bacterial loads than that observed for GFP UPs. Surprisingly, IFN-γ IBs showed a significant effect, diminishing bacterial loads by almost 6 times when compared to GFP IBs and by 11 times compared to the control. The IB format for the GWH1 carrier proteins, GWH1-IFN-γ and GWH1-GFP, showed a significant effect over bacterial loads in control glands, causing a decrease of 6.4 and 5.2 times, respectively. However, for these two samples only a numerically decrease in bacterial loads by 3.2 and 2.7 times in comparison to GFP IBs was observed, respectively.

**Figure 7:**
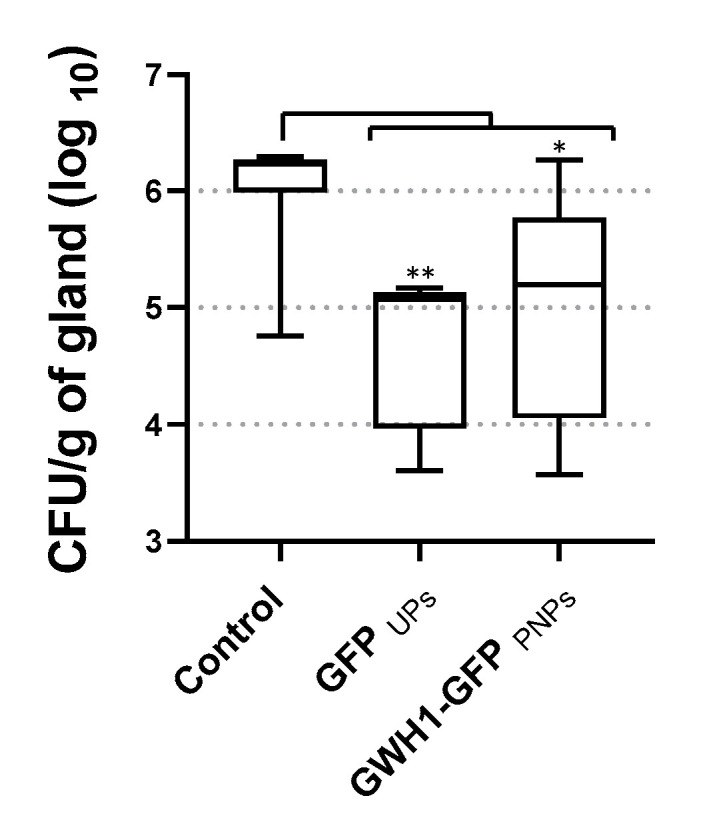
Antimicrobial effect of GFP and GWH1-GFP (PNPs) over *S. aureus*-infected mammary glands in vivo. Mammary glands were harvested and processed 8 h after the simultaneous administration of 100 CFU of *S. aureus* and 60 µmol/L of protein, respectively. Sodium bicarbonate buffer (166 mmol/L NaHCO_3_, 333 mmol/L NaCl) was used as control. The middle bars indicate median values for each group of glands (at least 6 glands) whereas the boxes specify quartiles Q1-Q3. The experiment was performed in triplicates. Significant results are shown as * *p* ≤ 0.05, ** *p* ≤ 0.01, one-way analysis of variance (ANOVA), followed by Tukey’s multiple comparisons test.

The performance of the GWH1-GFP construct forming PNPs prompted us to expand our in vivo study in *S. aureus*-challenged mice. In that case, GWH1-GFP PNPs caused a significant decrease in bacterial loads, showing 3.3 times less bacteria within the glands than the control (Figure 7). However, there was no difference when compared to the GFP UPs treatment, which also reduced *S. aureus* colonization.

## 4. Discussion

Novel therapies are urgently needed to tackle the reduced cure rates related to the development of bacterial resistance [29]. One alternative includes the use of alpha-helical AMPs [30]. However, large-scale production of synthetic small peptides is challenging [31] and recombinant protein production in prokaryotic host is presented here as a promising alternative. Several experimental strategies have been explored to cope with the inherent toxicity of such peptides, when recombinantly produced, towards the prokaryotic host, including the fusion to partner proteins that seem to mask the antimicrobial activity [32,33,34], as well as the accumulation in the insoluble cell fraction of AMPs fused to scaffold proteins [35]. In any case, regardless of the mechanism of action of alpha-helical AMPs against microorganisms, the recombinant production of these peptides might be achieved by the neutralization of their net charge by fusion to solubility-enhancing proteins [36,37,38] or by enhancing formation of IBs in producing cells by the fusion to aggregation-prone proteins [39].

In the present work, we have designed GWH1-containing recombinant proteins, with the idea of building bifunctional molecules where each individual part of the recombinant fusion design could maintain its original function, and at the same time, act as a building block for protein self-assembling [8,10]. In that sense, it has been demonstrated that GFP, toxins and pro-apoptotic proteins are able to act as scaffold partners for protein self-assembling while retaining their biological activity [9,40]. Protein production was affected by the expression of these AMP carrying proteins. The addition of GWH1 provoked a drastic decrease in protein yields in comparison to the non-AMP-containing counterparts, see Supplementary Table S1. However, proteins were produced in enough amounts to allow an effective purification process. Still, in the tested conditions, GWH1-IFN-γ arrangement was unable to promote the formation of soluble PNPs (Figure 1D), suggesting the inability of the mouse IFN-γ to act as a scaffold domain for protein oligomerization. Mature mouse IFN-γ forms noncovalently linked homodimers of 20–25 kDa [41]. The formation of dimers in both, IFN-γ and GWH1-IFN-γ could explain the bigger sizes observed by DLS and the bigger size of GWH1-IFN-γ over IFN-γ due to the presence of the GWH1 peptide. Moreover, the N-terminal addition of the GWH1 antimicrobial peptide has somehow diminished or truncated the mouse IFN-γ functionality as observed when comparing the activity of the IFN-γ and GWH1-IFN-γ proteins (Figure 3B). This fact is not surprising, since there is evidence that the N-terminus region of mouse IFN-γ plays an important role in receptor binding. Therefore, the failure to bind prevents internalization and later events that result in the induction of the immune response [42,43].

Surprisingly, antimicrobial performance of GWH1-containing proteins in different hosts, showed an inhibitory activity against *E. coli,* whereas *S. aureus* was more resistant to the treatment (Figure 2A,B). The different membrane composition may influence the mechanism of action of α-helical AMPs, especially for Gram-negative and Gram-positive bacteria [44]. In this sense, the GWH1 constructs produced in this work, not only have displayed a preferred antimicrobial activity against the Gram-negative *E. coli*, but, most importantly, the antimicrobial performance in this microorganism was enhanced when the GWH1-fusion proteins formed nanoparticles (Figure 2A). The main mechanism of action of AMPs is membrane permeabilization and structural disruption. To achieve the antimicrobial effect, a minimal AMP concentration is required in the target surface. Such characteristic is described as the threshold concentration [45] and is experimentally expressed as the peptide-to-lipid ratio (P/L). The propensity of GWH1-GFP to self-assemble in multimeric complexes could enhance the proximity of effective monomeric units on cell surface, diminishing the local peptide concentration required to reach higher P/L values. As the P/L ratio increases, the peptides start to insert and traverse the membrane. AMP monomeric forms require higher concentrations to achieve threshold concentrations. This was demonstrated in the case of the monomeric GWH1-GFP, which lacked a dose-dependent antimicrobial activity, only showing antimicrobial effect at the highest concentration, suggesting the need to reach a critical concentration in order to display its activity (Figure 2D). A similar result was observed with the unassembled GWH1-IFN-γ (Figure 2A). The presented evidence validates the multimeric format as a more effective arrangement than the monomeric format, when it comes to antimicrobial activity.

In order to explore the influence of the multiple display configuration of AMPs in the in vivo settings, we analyzed the performance of the engineered protein nanoparticles in a mouse mastitis model. Infectious mastitis is one of the most relevant diseases in dairy cattle, and antibiotic usage is the leading strategy for its treatment and prevention, since this disease is the costliest for dairy producers [46]. Despite the high antimicrobial activity showed by the multimeric format of GWH1-GFP, in vitro assays do not always correlate with in vivo efficacies inside the mammary gland [47,48]. Various aspects are influenced by the complex milk environment [25], and, therefore, a proof of concept in vivo approach is necessary. To date, different studies have used mouse models to assess the effect of antimicrobials [48,49,50,51]. However, most of these studies were dedicated to the Gram-positive pathogen *S. aureus* [25] and only few of them characterized the efficacy of antimicrobial agents on other relevant mastitis-causing pathogens such as *E. coli* [52,53]. To the best of our knowledge, this is the first study in which the antimicrobial capacity of AMP-containing nanoparticles has been determined in an in vivo mouse mastitis model of *E. coli* infection. Based on the in vitro assays for GWH1-GFP PNPs, a single dose of 60 µmol/L, i.e., ten times the MIC measured in vitro for GWH1-GFP in *E. coli* (Figure 2A), was selected for the intramammary administration after the bacterial challenge. As a result, the GWH1-GFP PNPs further reduced by 1-Log the bacterial burden in glands when compared with the non-AMP-containing counterpart, GFP (Figure 6A). This difference can be explained by the presence of the GWH1 antimicrobial peptide, which validates the functionality of this format in the milk environment and its efficacy as a promising anti-mastitis candidate. However, in *S. aureus* challenged animals, GWH1-GFP PNPs reduced bacterial load at the same level as control GFP (UPs) (Figure 7). In accordance with this, antimicrobial activity of GWHI-GFP PNPs in in vitro experiments against *S. aureus* was lower than that observed against *E. coli* (Figure 2A). As previously mentioned, the differences in the structure and physical properties of the bacterial membrane might account for the dissimilar activity of the same AMP in Gram-negative and Gram-positive bacteria [54]. On the other hand, in *E. coli*, GWH1-IFN-γUPs were not as effective as GWH1-GFP PNPs (Figure 6A), which supports the idea that the multimeric format is of utmost significance for reducing the local concentration of AMP necessary to achieve a deleterious membrane disruption, and, consequently, to increase the efficacy of the compound.

The IB format provides a protective environment against short-time degradation and a sustainable release of recombinant protein that may improve the effect over time [55,56,57]. However, as it has been described before [12], the amount of protein that can be released from IBs can vary depending on the IB-forming protein and the used experimental conditions during protein production process [58]. All this, associated with the fact that IFN-γ IBs have previously demonstrate activity, even at higher level than the soluble counterpart [12], urged us to test the efficacy of this format in an in vivo approach. The antimicrobial efficacy for GWH1-GFP IBs was partially reduced, showing a more disperse pattern which was not significant when compared to GFP IBs. This observation could be in accordance with the studied characteristics of GWH1-GFP IBs. The low availability of protein due to the poor release (Figure 1C) associated with the fact that part of the released protein can be found in a monomeric state (Figure 1E), which has been demonstrated here whereby bactericidal activity is reduced, may explain the low efficacy of this format in terms of antimicrobial activity. GWH1-IFN-γ and IFN-γ IBs slightly reduced the bacterial burden, being this latter statistically significant when compared to GFP IBs. This behavior could be associated with the increased release showed by both protein designs from IBs, especially for IFN-γ IBs (Figure 1C). Additionally, it is well known that IFN-γ has a very short half-life and therefore its immunological function in the mammary gland is limited [3]. The protective behavior provided by this oligomeric format may enhance its stability and merits further research. In fact, previous results showed that the activity of IFN- γ is preserved in protein released from IBs after a 96 h incubation at 37 °C [12]. The cytokine embedded in IBs could be less sensitive towards proteolytic degradation, which could then result in a better immunological performance inside the mammary gland. In fact, in the in vivo assays, the activity of the proteins containing IFN-γ displayed a higher activity when administered in IB format.

Altogether, the results presented in this work show a better antimicrobial performance of the multimeric AMP format against the monomeric configuration, and, most importantly, provided significant data for considering the AMP-containing nanoparticles as a promising alternative for treatment of mastitis. However, it should be noted that efficacy studies in mice should only be considered as an intermediate proxy to predict efficacy in dairy cows [51]. Overall, the versatility of the nanoparticle self-assembling format provides a valuable tool for testing a diversity of AMP with different scaffold proteins that could generate possible synergies or provide antimicrobial activity against other pathogens of interest.

## 5. Conclusions

The extensive use of antibiotics for the treatment and prevention of bacterial infections increases the selective pressure for appearance of resistant bacteria. Previously, we have described the antimicrobial activity of peptides fused to a scaffold protein and displayed in various copies on soluble PNPs. We have also analyzed the potential of naturally occurring protein supramolecular complexes, in particular IBs, to release biologically active soluble protein nanoparticles in in vivo approaches. In this work, we explored the relevance of the oligomeric state of antimicrobial peptides in such protein nanoparticles and studied their efficiency in vitro and in vivo against different mastitis causing pathogens. The results presented here indicate the potential of multiple displays of peptides in protein nanoparticles for the development of novel antimicrobial peptide-based formulations.

## Supplementary Materials

The following are available online at https://www.mdpi.com/1999-4923/12/12/1217/s1, Table S1: Solubility and productivity of recombinant proteins produced in the study. Soluble versions were produced at 20 °C for 5 h and IBs were produced at 37 °C for 3 h. Values represent mean and SEM when available. Not determined (n.d.).
