# Supplementary material for: In Vivo Bactericidal Efficacy of GWH1 Antimicrobial Peptide Displayed on Protein Nanoparticles, a Potential Alternative to Antibiotics
Source: Pharmaceutics. 2020 Dec 17;12(12):1217. doi: 10.3390/pharmaceutics12121217 (PMC7766456; doi:10.3390/pharmaceutics12121217)
Supplement: Supplementary file 1 [file pharmaceutics-12-01217-s001.pdf]

pharmaceutics

Supplementary Materials: In Vivo Bactericidal Efficacy of GWH1 Antimicrobial Peptide Displayed on Protein Nanoparticles, a Potential Alternative to Antibiotics

Jose V. Carratalá, Eric Brouillette , Naroa Serna, Alejandro Sanchez-Chardi, Julieta M. Sanchez, Antonio Villaverde, Anna Arís, Elena Garcia-Fruitós, Neus Ferrer-Miralles * and François Malouin *

Table S1. Solubility and productivity of recombinant proteins produced in the study. Soluble versions were produced at 20 °C for 5 h and IBs were produced at 37 °C for 3 h. Values represent mean and SEM when available. Not determined (n.d.).

Protein name	Soluble version (20 °C)		IBs (37 °C)	
	Solubility (%)	Productivity (mg/L)	Solubility (%)	Productivity (mg/L)
IFN-γ	60.20	26.6 ± 2.40	69.20	48.48 ± 15.73
GWH1-GFP	63.60	3.26 ± 0.81	16.90	13.40 ± 11.66
GWH1- IFN-γ	62.80	1.80 ± 1.92	51	22.67 ± 18.10
GFP	80.50 ± 9.68	92	n.d.	101.18 ± 27.29
